# Advances in Immunotherapies for Targeting Cancer Stem Cells in a Tumor Microenvironment: Emerging Strategies and Clinical Prospects

**DOI:** 10.3390/cells15100910

**Published:** 2026-05-15

**Authors:** Nakyung Oh, Van Ngu Trinh

**Affiliations:** 1 Department of Epigenetics and Molecular Carcinogenesis, University of Texas MD Anderson Cancer Center, Houston, TX 77030, USA; noh@mdanderson.org; 2 Center for Translational Cancer Research, Institute of Biosciences and Technology, Texas A&M University, Houston, TX 77030, USA

**Keywords:** cancer stem cells, tumor microenvironment, immunotherapy, immune checkpoint inhibitors, cancer vaccines, monoclonal antibody, nanobodies, bispecific antibody, antibody-drug conjugates (ADCs), CAR-T, CAR-NK

## Abstract

Cancer stem cells (CSCs) are a distinct subpopulation within a tumor that play an important role in tumor initiation, metastasis, therapeutic resistance, and cancer relapse. Their persistence is strongly influenced by the tumor microenvironment (TME), which provides a range of biological signals that maintain stemness, promote immune evasion, and resistance to cancer treatment. Therefore, effective targeting of CSCs is essential to improve therapeutic efficacy. In this review, we summarize the key characteristics of CSCs and their niche within the TME, emphasizing their interactions with immune cells, stromal components, and secreted factors. We also discuss the major challenges in targeting CSCs, including immune evasion, metabolic constraints, and intratumoral heterogeneity. We further highlight current and emerging immunotherapeutic strategies targeting CSCs, including immune checkpoint inhibitors, cancer vaccines, monoclonal antibodies, nanobodies, bispecific antibodies, antibody-drug conjugates (ADCs), CAR-T and CAR-NK cell therapies, oncolytic viruses, as well as innovative approaches such as targeted protein degradation. Finally, we emphasize the importance of a combinatorial approach that integrates CSCs targeting with modulation of the TME. Together, these strategies may lead to more durable responses, enhance therapy efficacy and reduce the risk of tumor recurrence.

## 1. Introduction

Cancer stem cells (CSCs) are a subpopulation of tumor cells, characterized by their capacity for self-renewal, differentiation, and tumor initiation [1]. CSCs were first identified in hematological malignancies [2,3], and subsequently in solid tumors [4,5,6,7,8,9,10,11]; they are now recognized as a core component of tumor initiation. CSCs also contribute to multiple hallmarks of cancer, including tumor heterogeneity and promoting metastasis through epithelial–mesenchymal transition (EMT) [12,13]. Beyond the intrinsic characteristics CSCs acquire from their cell fate, their survival and function are further supported by a complex ecosystem known as the tumor microenvironment (TME). In vivo, CSCs reside within the TME, which further reinforces key CSC features. For example, the hypoxic nature of TME enhances cancer invasiveness and dissemination [14]. In addition, under hypoxic conditions within the TME, CSCs can enter a dormant state and later reinitiate proliferation when the surrounding TME becomes favorable. In clinical settings, this switch in cell cycle contributes to disease relapses after initial treatment [15,16].

Because of these features, CSCs appear to be ideal candidates for therapeutic targeting, as their elimination could, in principle, lead to a complete eradication of the tumor. However, significant challenges remain in effectively targeting and eradicating these cells, largely due to another defining feature of CSCs, which is their innate therapeutic resistance. CSCs employ multiple mechanisms to evade conventional therapies, including enhanced DNA damage repair capacity, activation of anti-apoptotic signaling pathways, increased expression of drug efflux transporters, and metabolic plasticity [17]. Additionally, their quiescent state reduces susceptibility to therapies that primarily target rapidly dividing cells [18,19]. Therefore, developing more effective therapeutic strategies requires a comprehensive understanding of CSC biology, including their role in cancer progression, their complex interactions with the TME, and the mechanisms underlying their treatment resistance. Ultimately, targeting CSC-specific pathways and overcoming these resistance mechanisms may offer a promising approach to improving long-term clinical outcomes and reducing cancer recurrence.

### 1.1. CSCs in the TME

The TME is composed of diverse cellular components, including cancer-associated fibroblasts (CAFs), immune cells, and endothelial cells, as well as the extracellular matrix (ECM), alongside non-cellular factors such as cytokines, growth factors, and hypoxic gradients. These components interact with CSCs through complex signaling networks that regulate their behavior and functional plasticity [20].

Within the broader TME, CSCs preferentially reside in a highly dynamic and specialized region often referred to as the CSC niche, which plays a crucial role in maintaining their stemness, survival, and therapeutic resistance. CSCSs derive multiple benefits from this niche, with hypoxia being a key example [21]. Indeed, CSCs are often located adjacent to hypoxic areas within the tumor [22]. Hypoxia activates transcriptional programs that promote stemness [23], metabolic reprogramming [24], and resistance to apoptosis [25]. Importantly, CSCs are not merely passive residents that benefit from the TME; rather, they actively remodel their surrounding microenvironment by secreting factors such as vascular endothelial growth factor (VEGF), transforming growth factor-β (TGF-β), and interleukins, thereby promoting a pro-survival microenvironment [26]. However, not all components of the TME are beneficial to CSCs. Some pose significant threats. Among the most prominent are cytotoxic immune cells, which patrol the body to identify and eliminate cancer cells. To evade immune surveillance, CSCs have developed a range of escape mechanisms, such as downregulation of antigen presentation machinery [27], upregulation of immune checkpoint molecules [28], and secretion of inhibitory cytokines that impair cytotoxic T cell and natural killer (NK) cell function [29]. Additionally, CSCs can recruit and polarize immune cells such as tumor-associated macrophage (TAMs) and regulatory T cells (Tregs), further reinforcing an immunosuppressive microenvironment and promoting tumor progression [30]. Another component of TME, stromal cells, particularly CAFs, play a pivotal role in sustaining CSC phenotypes [31]. Through the secretion of signaling molecules such as Wnt ligands [32], Notch ligands [33], and hepatocyte growth factors (HGFs) [34], CAFs enhance CSC self-renewal and promote EMT. In addition, the ECM contributes to this regulation by providing structural support and biochemical cues that influence CSC behavior, including stiffness-mediated signaling and integrin activation [35].

Overall, the bidirectional interactions between CSCs and components of the TME create a supportive niche that not only preserves CSC properties but also enhances tumor progression, metastasis, and therapeutic resistance. Therefore, targeting these interactions within the CSC niche represents a promising strategy for disrupting tumor growth and improving therapeutic efficacy.

### 1.2. Objective and Scope of the Review

CSCs are increasingly recognized as key drivers of tumor heterogeneity and resilience. However, their behavior is profoundly shaped by the specialized niches they inhabit. Therefore, this review first introduces the fundamental biological properties of CSCs, followed by an in-depth discussion of the CSC niche within the TME, highlighting the dynamic interplay between CSCs and stromal, immune, and extracellular components. In addition, this review examines the major challenges associated with targeting CSCs in the context of the TME, including immune evasion mechanisms and the protective effects of the microenvironment, which collectively limit the efficacy of conventional therapies.

Finally, we discuss emerging immunotherapeutic strategies, including immune checkpoint blockade, adoptive cell therapies, cancer vaccines, and combination approaches aimed at overcoming CSC-associated resistance. By integrating insights into CSC biology, niche-specific regulation, and therapeutic vulnerabilities, this review aims to highlight potential avenues for improving cancer treatment efficacy and to provide a framework for the development of CSC-directed immunotherapies.

## 2. Characteristics of Cancer Stem Cells

### 2.1. Defining Features of CSCs

CSCs represent a small subpopulation of tumor cells with several unique features that contribute to cancer initiation, progression, relapse, and therapy resistance. The most distinctive characteristic of CSCs is their self-renewal capacity, which allows them to undergo indefinite division and generate additional CSCs, thereby maintaining the tumor-initiating population, similar to normal stem cells [1]. Tumorigenicity is another key property of CSCs [36]. Owing to their highly tumorigenic nature, as few as 100–200 CSCs can be sufficient to reinitiate tumor formation [4,5]. Moreover, although chemotherapy and radiotherapy can effectively reduce the bulk of the rapidly proliferating tumor cells, CSCs often survive due to their quiescent state and retain the ability to regenerate the tumor, ultimately leading to disease relapse [36]. In addition, when CSCs give rise to tumors, they generate diverse populations of cancer cells, resulting in intratumoral heterogeneity. This hierarchical organization enables tumors to sustain long-term growth while simultaneously producing rapidly proliferating cells that constitute the bulk of the tumor [37]. Furthermore, CSCs are considered relatively immune-privileged and can actively modulate the surrounding immune microenvironment through interactions with both cellular and acellular components of the TME, like stromal cells, immune cells, and the extracellular matrix [38]. These interactions will be discussed in further detail in a subsequent section of this article. Beyond these properties, CSCs can dynamically switch their phenotype from an epithelial to a mesenchymal state through a process known as EMT. EMT enables CSCs to acquire enhanced motility and invasiveness, facilitating their dissemination, survival in circulation, and colonization of distant sites to seed new tumorigenic growth [39,40]. Taken together, these features highlight CSCs as key drivers at all stages of disease progression and underscore their importance as therapeutic targets for cancer treatment.

### 2.2. Identified Markers of CSCs

The identification of CSCs relies on a combination of functional assays and molecular markers, although no universal marker exists across all cancer types. Commonly used markers include CD133 (prominin-1) [5,41], CD44 [1], CD24 [42,43], epithelial cell adhesion molecules (EpCAM) [14], and aldehyde dehydrogenase (ALDH) 1 activity [44], which are variably associated with stem-like properties across malignancies. For example, CD44^high^/CD24^low^ phenotypes are frequently used to identify CSCs in breast cancer [4], while CD133 expression has been widely reported in the brain [5,41], colorectal [45], and pancreatic cancers [46]. Importantly, CSC marker expression is heterogeneous even within the same cancer type. For instance, in glioblastoma multiforme, CSC identification is not restricted to CD133^+^ populations, as CD133^−^ cells also exhibit tumor-initiating capacity [47]. Moreover, CSCs expressing ganglioside D3 (GD3) and its synthase enzyme, GD3 synthase, show strong associations with enhanced stemness, self-renewal, and tumorigenicity, independent of CD133 expression [48]. Similarly, glioblastoma CSCs expressing A2B5, a cell surface ganglioside, also display stem cell-like properties even in the absence of CD133 expression [49]. In addition, high ALDH activity, commonly assessed using ALDEFLUOR assays, is frequently used as a functional marker, as ALDH activity correlates with self-renewal and chemoresistance [50].

CSCs are also characterized by the activation of key developmental signaling pathways that regulate stemness and differentiation (Wnt/β-catenin, Notch, and Hedgehog pathways) [51]. In addition, transcription factors (OCT4, SOX2, and NANOG) contribute to the maintenance of pluripotency-like states in CSCs [52,53]. However, the expression of these markers and signaling pathways can vary significantly depending on tumor type, stage, and microenvironmental context, which complicates their use as definitive CSC identifiers [1]. Given this heterogeneity, robust CSCs identification requires integration of phenotypic markers with functional assays, including sphere formation assays [54], lineage tracing [55], and in vivo tumorigenicity assays [56]. Recent advances in single-cell sequencing and spatial transcriptomics are further refining CSC identification by resolving stem-like populations within the tumor at high resolution [57]. Collectively, these approaches highlight both the complexity and the evolving nature of the CSC definition, underscoring the need for context-dependent strategies to accurately identify and target CSCs in cancer (Table 1).

### 2.3. CSC-Intrinsic Mechanisms Driving Immune Evasion and Treatment Failure

CSCs remain one of the most challenging tumor populations to eradicate, owing to their exceptional capacity to survive under both therapeutic and immunological pressures [130,131]. Unlike most tumor cells, CSCs possess a range of intrinsic properties that enable them to persist despite aggressive treatments, ultimately contributing to tumor relapse and disease progression. In this section, we focus specifically on the innate cellular programs of CSCs and discuss how they evade immune-mediated elimination and survive against conventional therapies. We also briefly address the heterogeneous nature of the CSC population.

#### 2.3.1. Immune Tolerance

CSCs possess an “immune-privileged” phenotype and can evade both innate and adaptive immune responses, primarily through defective MHC-I and MHC-II expression. The loss or reduction in these molecules impairs effective antigen presentation to T cells, thereby limiting immune recognition [38]. In CSCs, stemness-associated signaling pathways are closely linked to this immune-evasive machinery. For instance, activation of the Wnt/β-catenin signaling pathway, which is frequently implicated in maintaining CSC properties, has been shown to suppress MHC expression in brain tumor CSCs, thereby reducing immunogenicity [132]. In addition, core pluripotency transcription factors such as NANOG and OCT4 are associated with transcriptional repression of MHC-related genes, further contributing to an immune-invisible phenotype that supports tumor persistence and resistance to immunosurveillance [133].

However, immune privilege alone is insufficient for complete immune escape, as CSCs express immunogenic tumor-associated antigens (TAAs) such as carcinoembryonic antigen (CEA), human telomerase reverse transcriptase (hTERT), survivin, and some cancer testis (CT) antigens, including DnaJ homolog subfamily B member 8 (DNAJB8), and mucin 1 (MUC1) [134,135]. To overcome immune pressure, CSCs rely heavily on their surrounding microenvironment, referred to as the CSC niche. CSCs actively shape an immunosuppressive TME by secreting cytokines such as TGF-β and IL-1 which inhibit effector T cell activity, and by recruiting and polarizing immunosuppressive cell populations such as monocytes, myeloid-derived suppressor cells (MDSCs), TAMs, and Treg [136]. CSCs, together with stromal cells, further reinforce this immunosuppressive niche through the secretion of inhibitory factors, promotion of suppressive immune cell populations, and enhancement of CSC stemness via paracrine signaling [137]. Further details regarding CSC-niche interactions and niche reprogramming will be discussed in Section 3.2.

Additional immune escape mechanisms include the dynamic regulation of various cell surface proteins. For instance, CSCs upregulate CD47, a well-known “don’t eat me” signal, while downregulating proteins involved in innate immune pathways (e.g., toll-like receptor 4 (TLR4) and signal transducer and activator of transcription 3 (STAT3) pathway) [138,139,140,141]. CSCs also exploit immune checkpoint pathways to evade immune cell-mediated killing. A prominent example is programmed death-ligand 1 (PD-L1), an immune checkpoint ligand that is often highly expressed in CSCs. PD-L1 binds to its receptor PD-1 on activated effector T cells, delivering an inhibitory “off” signal that suppresses T cells’ cytotoxic function [142]. Notably, CSCs exhibit elevated expression of immune checkpoint molecules due to crosstalk between stemness-associated signaling pathways and immune regulatory programs, as these pathways broadly reprogram gene expression. In the Wnt/β-catenin pathway, nuclear β-catenin forms a complex with TCF/LEF transcription factors, which bind to promoters and enhancer regions of the PD-L1 gene and directly enhance its transcription [143]. In the Notch signaling pathway, activation leads to the release of the Notch intracellular domain (NICD), which translocates to the nucleus and functions as a transcriptional co-activator by binding to regulatory regions of the PD-L1 [144]. Similarly, activation of the Hedgehog pathway induces GLI1/2 transcription factors, which bind to PD-L1 regulatory elements and promote its expression [145].

#### 2.3.2. Therapy Resistance and Heterogeneity of CSCs

CSCs tend to be more resistant to conventional chemotherapy and radiotherapy due to their robust DNA damage repair machinery and their ability to enter a dormant or anti-apoptotic state [146,147,148]. In addition, CSCs express high levels of ATP-binding cassette (ABC) transporters, which efflux cytotoxic compounds from the cell, further contributing to treatment resistance [149,150].

Beyond these intrinsic resistance mechanisms, heterogeneity within the CSC population further enhances therapy resistance [151]. Tumor heterogeneity is a fundamental hallmark of cancer and is particularly pronounced within CSC populations. Rather than representing a uniform subpopulation, CSCs exhibit substantial phenotypic and functional diversity both within and across tumor types. This heterogeneity arises from genetic mutations, epigenetic modifications, and dynamic interactions with the TME [152]. As a result, distinct CSC subsets may express different surface markers (e.g., CD44, CD133, and ALDH1), display variable proliferative capacities, and rely on divergent signaling pathways for self-renewal [153]. Importantly, CSCs exist along a continuum of cellular states, ranging from quiescent, therapy-resistant cells to more proliferative progenitor-like populations [154]. Moreover, cellular plasticity further complicates this landscape, as non-stem cancer cells can dedifferentiate into CSC-like states under specific microenvironmental cues, highlighting that “stemness” is not a fixed trait but rather a dynamic and reversible condition [155,156].

## 3. The CSC Niche and Tumor Microenvironment

### 3.1. The CSC Niche

The CSC niche is a specialized and highly regulated sub-compartment within the TME that specifically supports the maintenance, self-renewal, and survival of CSCs [20]. Compared with the broader TME, the CSC niche is more functionally selective, being enriched in environmental factors such as hypoxic conditions, specific cytokines, and direct cell–cell interactions with niche-supporting cells like CAFs or endothelial cells. Collectively, these cellular and acellular components engage in bidirectional interactions with CSCs to sustain tumor progression and adaptation [131]. In contrast, the general TME primarily supports overall tumor growth, invasion, and immune evasion without necessarily maintaining stem-like properties. Despite these distinctions, the CSC niche and the TME share many overlapping features. Both are shaped by dynamic interactions between tumor cells and surrounding stromal and immune components, and both rely heavily on key signaling pathways such as Notch [157], Wnt/β-catenin [158], and Hedgehog [159]. Additionally, processes like angiogenesis [160], immune modulation [161], and ECM remodeling [162] are common to both contexts. Importantly, the CSC niche does not exist independently but is embedded within the broader TME; therefore, alterations in the TME, such as inflammation or therapeutic intervention, can directly influence CSC behavior. Thus, while the CSC niche represents a more specialized microenvironment, it is fundamentally interconnected with and dependent on the larger tumor ecosystem.

The CSC niche consists of diverse and spatially organized populations of stromal and immune cells that vary across tumor regions and disease stages [163]. CAFs, for example, are not a single homogeneous entity but comprise multiple subtypes with distinct transcriptional profiles and functions, including inflammatory CAFs (iCAFs), which secrete cytokines, and myofibroblastic CAFs (myCAFs), which contribute to ECM deposition and tissue stiffness [164]. Similarly, TAMs exist along a continuum of activation states rather than discrete M1/M2 categories, with different subsets exerting either pro-inflammatory or immunosuppressive functions depending on local microenvironmental cues [165]. The abundance and functional states of other immune cells, including T cells, dendritic cells (DCs), and NK cells, also vary widely, contributing to regional differences in immune activity within the same tumor [166]. This spatial and cellular diversity generates distinct micro-niches with unique biochemical and mechanical properties.

Crucially, CSC heterogeneity is tightly linked to the heterogeneity of the CSC niche, as distinct microenvironmental niches selectively support specific CSC states [167]. Hypoxic regions, for instance, tend to enrich quiescent, stem-like cells with enhanced resistance to therapy, whereas perivascular niches provide access to oxygen, nutrients, and endothelial-derived signals that promote CSC proliferation and self-renewal [168]. Stromal and immune cells within these niches secrete distinct combinations of cytokines, growth factors, and extracellular vesicles that shape CSC behavior in a context-dependent manner [169]. Moreover, spatial gradients of oxygen, pH, and nutrient availability across the tumor further diversify CSC phenotypes by inducing metabolic and transcriptional adaptations [170]. These bidirectional interactions ensure that CSCs and their niche co-evolve, reinforcing intratumoral heterogeneity and functional specialization.

This extensive heterogeneity has significant implications for therapeutic intervention, as it underlies variable treatment responses and contributes to disease progression and relapse. Therapies targeting a single CSC population or a specific component of the CSC niche may fail due to the presence of alternative CSC states or compensatory microenvironmental mechanisms. Moreover, treatment itself can reshape both CSC populations and the TME, often selecting for more aggressive and therapy-resistant phenotypes [171]. A comprehensive understanding of this dynamic and heterogeneous ecosystem is therefore essential for the development of effective therapeutic strategies. To this end, approaches integrating single-cell profiling, spatial analysis, and systems biology are increasingly being employed to map CSC and TME diversity, providing new opportunities to design combination therapies capable of overcoming heterogeneity and improving clinical outcomes [172,173].

### 3.2. Crosstalk Within the CSC Niche: CSC Maintenance, Immune Evasion, and Therapy Resistance

#### 3.2.1. Immune Cells

CSCs actively shape an immunosuppressive tumor microenvironment through the secretion of more immunosuppressive cytokines and less pro-inflammatory cytokines, as well as through the recruitment and activation of immunosuppressive cell populations such as Tregs, MDSCs, and TAMs, together with the inhibition of cytotoxic T cell and NK cell function. This process is facilitated by key pluripotency-associated transcription factors such as SOX2, OCT4, and NANOG, which extend beyond their canonical roles in maintaining CSC self-renewal to actively contribute to immune modulation. For instance, OCT4 and SOX2 can cooperatively induce an immunosuppressive transcriptome characterized by the upregulation of immune checkpoint molecules such as PD-L1, CD70, A2aR, and TDO, along with broad dysregulation of cytokines and chemokines that promote a suppressive tumor microenvironment [174]. Similarly, NANOG has been reported to downregulate pro-inflammatory cytokines, such as IL-1β, TNF-α, and IL-6 at both mRNA and protein levels through suppression of NF-κB activity, thereby further dampening inflammatory response in favor of immune evasion [175]. Once immunosuppressive immune cells like TAMS and Tregs are recruited, CSCs can reprogram them into phenotypes that support immune tolerance, tissue remodeling, and tumor progression [176]. These reprogrammed cells, in turn, secrete factors that reinforce CSC stemness and survival, establishing a feed-forward loop that sustains both immune evasion and CSC maintenance.

As discussed in the previous section (Section 2.3), the upregulation of immune checkpoint molecules represents a major mechanism of immune evasion in CSCs. CSCs frequently exhibit elevated expression of PD-L1, CTLA-4, TIM-3, and LAG-3, which collectively contribute to the induction of dysfunctional T cell states. Ultimately, chronic antigen exposure from CSCs, combined with persistent inhibitory signaling, drives epigenetic and transcriptional reprogramming of T cells toward an exhausted phenotype that is resistant to immune-mediated clearance [177]. Moreover, in addition to stemness-induced PD-L1 expression, interferon signaling within the TME can paradoxically enhance PD-L1 levels, thereby reinforcing a self-amplifying immunosuppressive loop [178].

Metabolic reprogramming within the TME represents another critical axis of immune evasion. Rapidly proliferating tumor cells rely heavily on glycolysis, leading to glucose depletion, lactate accumulation, and acidification of the local microenvironment. This metabolic competition impairs effector T cells and NK cells’ function by depriving them of essential energy sources, while lactate directly suppresses their cytotoxic activity and cytokine production [179]. In parallel, indoleamine 2,3-dioxygenase (IDO), which catabolizes tryptophan into kynurenine, promotes Treg differentiation and suppresses effector T cell responses through kynurenine. Additionally, adenosine accumulation, driven by ectonucleotidases, CD39 and CD73 expressed on tumor and stromal cells, further inhibits immune activation via A2A receptor signaling [180,181]. Interestingly, these metabolic conditions are particularly favorable for CSC survival, as CSCs are metabolically flexible and adapt better to hypoxic and nutrient-deprived environments compared to tumor cells [182].

CSCs can also evade immune surveillance by adopting features that mimic immune cells, thereby blurring the distinction between malignant and normal immune populations. Through transcriptional reprogramming, CSCs may express surface markers and signaling molecules typically associated with immunosuppressive cells such as Treg or MDSCs. This immune cell mimicry enables CSCs not only to avoid immune recognition but also to actively contribute to the establishment of an immunosuppressive milieu. This deceptive strategy allows CSCs to “hide in plain sight”, functioning in ways that resemble physiological immune regulation while simultaneously protecting themselves from immune-mediated elimination [183].

In addition, immunosuppressive immune cells like TAMs, MDSCs, and Tregs contribute to therapy resistance by secreting cytokines like IL-6, which activate STAT3 signaling in cancer cells. This signaling axis promotes CSCs enrichment and upregulation of stemness-associated transcription factors such as SOX2, OCT4, and NANOG [184,185]. These cytokine-driven pathways maintain CSC properties, enhance cellular plasticity, and ultimately contribute to therapeutic resistance [186]. Furthermore, chemotherapy does not exclusively target cancer cells but also significantly alters components of the TME. Emerging evidence indicates that taxane-based chemotherapeutic agents can reprogram macrophages within the TME, leading to increased expression of cathepsin proteases that protect tumor cells from chemotherapy-induced cell death. This highlights an unintended pro-tumor effect of chemotherapy mediated by non-malignant components of TME, particularly macrophages [187,188].

#### 3.2.2. Stromal Cells

CSCs actively establish and maintain the CSC niche by recruiting supporting stromal cells, including vascular endothelial cells, fibroblasts, and mesenchymal stem cells (MSCs) from nearby stroma and bone marrow [189]. Once recruited, these cells interact with CSCs and endothelial cells, leading to the reprogramming of normal fibroblasts into CAFs [20]. CSCs further activate CAFs through microRNA-containing exosomes and pro-fibrotic cytokines such as TGF-β and interferon regulatory factor 6 (IRF6), thereby promoting ECM remodeling and niche stabilization [190,191,192,193].

Among stromal components, CAFs and MSCs represent key regulators of the CSC niche [194]. CAFs secrete a wide array of growth factors, cytokines, and extracellular vesicles such as TGF-β, CCL2, and CXCL-12 to enhance CSC self-renewal [195,196], IL-6 and IL-8 to promote EMT [197], and POSTN to support stemness and metastatic colonization in colorectal cancer [198]. In addition, CAF-derived ligands activate multiple signaling pathways in CSCs, including Wnt/β-catenin [199], Notch [200], yes-associated protein (YAP), integrins [201] and Hedgehog [202], collectively reinforcing stem-like phenotypes. MSCs also contribute to niche formation by secreting chemokines and immunomodulatory factors that support CSC maintenance and facilitate metastatic dissemination [203]. Furthermore, CSC-derived angiogenic factors stimulate the migration, proliferation, and lumen formation of endothelial progenitor cells, which in turn reinforce CSC self-renewal and tumorigenesis through the release of additional pro-angiogenic signals [204,205]. Within the perivascular niche, endothelial cells provide CSCs with oxygen, nutrients, and angiocrine signals that regulate stemness and quiescence [206]. CSCs also actively recruit MSCs [207,208], which upon stimulation by TGF-β in TME, upregulate Jagged-1, a Notch ligand associated with tumor metastasis, thereby promoting CSC stemness and EMT [209,210]. Additionally, cancer-associated MSCs can increase the expression of bone morphogenetic protein 2 (BMP2), further expanding CSC populations in primary ovarian cancer [211].

Beyond supporting CSC maintenance, MSCs and CSCs together reinforce the immunosuppressive nature of the niche by secreting inhibitory factors, promoting suppressive immune cell populations, and enhancing CSC stemness through paracrine signaling [141]. MSCs possess potent immunosuppressive properties that CSCs co-opt to inhibit the proliferation and function of T cells, B cells, and NK cells, while also modulating DCs’ activity and promoting Treg induction both in vivo and in vitro. These effects are mediated through direct cell–cell interactions and soluble factors such as IDO, prostaglandin E2 (PGE2), nitric oxide (NO), HLA-G, TGF-β, interferon (IFN)-Ɣ, and IL-1β. Through these mechanisms, CSCs enhance local immunosuppression, reduce immune activation, and establish a protective niche that facilitates immune evasion and tumor progression [203]. CSCs can further evade immune surveillance through a process of cellular mimicry, in which they adopt phenotypic and functional traits of certain immune-privileged cell types [183]. One well-characterized example is endothelial or vascular mimicry, where CSCs express endothelial-like markers and form vessel-like structures. By resembling normal vascular cells, which are typically tolerated by the immune system, CSCs reduce immune recognition and create physical barriers to cytotoxic immune cell infiltration [212,213]. In addition, extracellular vesicles derived from tumor and stromal cells carry immunosuppressive proteins, lipids, and microRNAs that reprogram recipient immune cells toward tolerogenic phenotypes [214]. Together, these mechanisms establish a multilayered immunosuppressive network that enables tumor persistence despite immune surveillance.

Importantly, CAFs and other stromal components also contribute to therapy resistance. CAFs secrete paracrine factors and exosomes that enhance tumor cell survival under therapeutic stress. For example, CAFs-derived IL-6 activates the JAK/STAT pathway, promoting resistance to gemcitabine [215], while CXCL12 activates JAK2/STAT3 pathway, inhibiting degrade of PD-L1 and conferring resistance to immunotherapy [216]. IL-8 further activates NF-κB and ABCB1 upregulation, contributing to cisplatin resistance in gastric cancer and non-small cell lung cancer [217,218]. In addition, CAF-mediated activation of MAPK and PI3K/AKT pathways through factors such as EGF, TGF-β, and IGF promote tumor survival, stemness, and reduced drug sensitivity. These signaling cascades are often co-activated, forming compensatory networks that enable escape from targeted therapies. CAFs also promote resistance through Wnt/β-catenin and Notch signaling, further reinforcing tumor progression, EMT, and therapy resistance [219]. CAFs-derived exosomes additionally transfer microRNAs that reprogram tumor cells and contribute to resistance. For example, miR-93-5p and miR-22 enhance proliferation and confer resistance to radiotherapy and endocrine therapy by targeting FOXA1, PTEN, and estrogen receptor signaling [220,221]. Similarly, miR-20a activates PI3K/AKT signaling via PTEN suppression, promoting cisplatin resistance in lung cancer [222]. CAF-derived microRNAs can also inhibit ferroptosis, such as miR-432-5p targeting CHAC1, further enhancing chemoresistance [223].

Tumor-associated endothelial cells (TECs) also contribute to drug resistance by promoting CSC survival and chemoresistance through angiocrine signaling. Under physiological conditions, TEC-derived IGFBP7 suppresses CSC expansion by inhibiting IGF signaling. However, chemotherapy disrupts this balance by reducing IGFBP7 and increasing IGF1 production. This shift activates IGF1R signaling in CSCs and triggers a feedforward FGF4–FGFR1–ETS2 axis, ultimately promoting CSCs maintenance, chemoresistance, and tumor progression [224]. Moreover, tumor neovasculature itself contributes to therapeutic failure, as it is highly abnormal, characterized by disorganized, immature, and hyperpermeable vessels with irregular structure and poor hierarchy. This chaotic architecture leads to uneven blood flow and inefficient drug delivery, thereby creating protective niches that enable CSC survival under therapy [225].

#### 3.2.3. ECM

In addition to cellular components, the ECM is a crucial element for CSC embedding within the TME. Rather than a passive scaffold, the ECM acts as an active regulator of CSC behavior by providing both structural and biochemical support [226]. ECM components like collagen, fibronectin, and laminin interact with CSCs through integrins and other adhesion molecules, thereby activating intracellular signaling pathways that influence proliferation, survival, and differentiation [227]. Within the TME, CAFs are the primary producers of ECM. CAFs secrete BMP1, thrombospondin-1, and elastin interface 2, as well as various splice variants of fibronectin ED-A and ED-B, and tenascins C and W. As a result, the tumor forms a stiff ECM compared with adjacent normal tissue [228,229,230].

The mechanical properties of the ECM, including stiffness and topography, modulate CSC phenotype through mechanotransduction pathways such as Rho/ROCK and YAP/TAZ [226]. CSCs respond to matrix stiffness differently in a context-dependent manner. In soft 3D fibrin matrices, melanoma CSCs showed increased self-renewal with histone H3 lysine 9 (H3K9) demethylation and higher SOX2 expression [231,232]. However, in breast CSCs, stiffer matrices increased CSC marker expression via integrin-linked kinase (ILK) signaling, suggesting that the influence of matrix stiffness is highly tumor-type dependent [233]. In addition, under hypoxic conditions, cancer cells, CAFs, and TAMs cooperatively remodel the ECM by increasing deposition of structural components such as collagens and cross-linking enzymes from the lysyl oxidase (LOX) and transglutaminase families [234,235]. As a result, collagen and elastin fibers become reorganized and extensively cross-linked by LOX and transglutaminase, forming larger and more rigid fibers that facilitate cell migration and invasion [236,237]. Compared with the broader TME, the ECM in the CSC niche is more spatially organized and enriched in stemness-regulating cues, enabling precise control of cell fate decisions and maintenance of CSC properties [226]. ECM topology further influences CSC self-renewal by regulating the balance between symmetric and asymmetric cell division. The way ECM is arranged in TME space can direct the orientation of the cell division axis by controlling where actin forms at the cell membrane through focal adhesions, as well as how cellular components are distributed during interphase [238].

Beyond its role in tumor cell regulation, the physical and structural properties of the ECM create substantial obstacles to immune cells [239,240]. Dense ECM deposition and stromal remodeling by CAFs restrict the access of cytotoxic immune cells to regions where CSCs reside, particularly within hypoxic and perivascular niches [241]. At the same time, the ECM can facilitate the recruitment of immunosuppressive cells like TAMs and Treg while paradoxically inhibiting the migration of antitumorigenic immune populations [242,243,244,245]. In addition, high collagen components inhibit the proliferation and activation of cytotoxic T cells via type I collagen-dependent fusion of Leukocyte-associated immunoglobulin-like receptor (LAIR) [246].

Finally, dense physical barriers of the ECM are one of the mechanisms of drug resistance. For example, cisplatin can bind to collagen fibers, decreasing drug penetration and thereby enhancing CSC survival. In addition, cell–matrix interactions, known as cell adhesion-mediated drug resistance, activate pro-survival signaling pathways in cancer cells and protect them from apoptosis [247].

#### 3.2.4. Hypoxia

The hypoxic nature of the TME in solid tumors induces the expression of stem cell markers and maintains CSCs in an undifferentiated state [248,249]. Under hypoxic conditions, cancer cells activate hypoxic-inducible signaling pathways, leading to the stabilization and upregulation of hypoxia-inducible factor (HIF)-1α and HIF-2α levels. Under normoxic conditions, HIF1α is rapidly degraded through ubiquitination. However, under low oxygen tension, HIF1α gets stabilized, translocated into the nucleus, and dimerizes with HIF1B protein to initiate the transcription of hypoxia-related genes as well as stemness-associated genes such as CD133, CD24, DLK1, and POU5F1, along with self-renewal-related transcription factors such as OCT4, NANOG, and SOX2 [1,250,251]. Importantly, HIF2α is more selectively expressed in CSC populations, whereas HIF1α is present in both stem and non-stem cancer cells under hypoxic conditions. Importantly, a reduction in HIF activity in CSCs leads to impaired survival, highlighting its functional relevance in maintaining stemness [252]. However, several important caveats should be considered. In solid tumors, hypoxia exists as a gradient within the TME, with relatively higher oxygen levels in perivascular regions and severe hypoxia in necrotic areas. Reflecting this heterogeneity, CSCs in brain tumors are found in at least two distinct niches: perivascular regions and areas surrounding necrotic tissue [167]. Hypoxia also prevents differentiation of cells within the CSC niche, such as MSCs, thereby helping maintain an undifferentiated state through interactions with immature stromal populations [253].

In addition to previously described canonical immune evasion mechanisms, hypoxia further exacerbates immune suppression by HIF-mediated regulation, including upregulation of immune checkpoint ligands, recruitment of suppressive immune cell populations, and inhibition of DCs maturation [254,255,256].

The hypoxic nature of TME also promotes drug resistance through inducing stemness and cellular quiescence [257]. Activation of HIF1α signaling reduced intracellular reactive oxygen species (ROS), thereby decreasing apoptosis in cancer cells [258]. In mammospheres, a structure enriched in stem cells, CSCs exhibit lower ROS levels than differentiated adherent cells, contributing to resistance against radiation therapy [259,260]. Similarly, oral cancer cells with reduced ROS levels, due to elevated expression of antioxidant enzymes such as catalase, SOD2, and peroxiredoxin 3, display enhanced CSC-like characteristics and greater resistance to cisplatin compared with ROS^high^ counterparts [261]. A key regulator of low ROS levels is ALDH, a well-established CSC marker [105]. ALDH enzymes detoxify intracellular aldehydes and participate in retinoic acid metabolism during early stem cell differentiation processes [262]. Among them, ALDH1 plays a major role by directly lowering ROS levels and generating antioxidant molecules such as NADPH, while also protecting cells from chemotherapeutic agents like paclitaxel [263]. Importantly, tumors with elevated ALDH activity tend to be more tumorigenic and resistant to chemotherapy, partly due to enhanced DNA repair capacity, highlighting its role in protecting CSCs from therapeutic stress [129].

**Figure 1:**
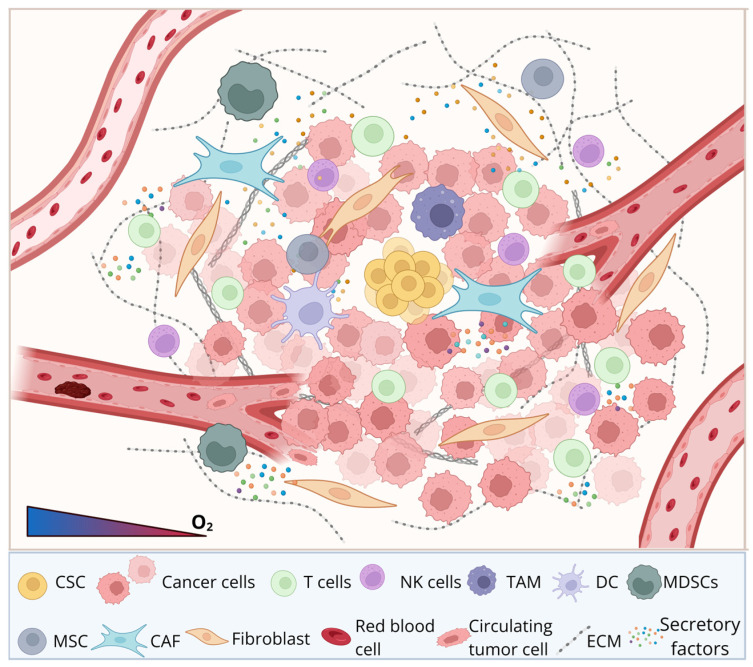
Cancer stem cell (CSC) niche in solid tumors. The CSC niche is a complex and dynamic microenvironment composed of heterogeneous cancer cells and CSCs, together with stromal components such as mesenchymal stem cells (MSCs), fibroblasts, and cancer-associated fibroblasts (CAFs), as well as immune populations including T cells, natural killer (NK) cells, dendritic cells (DCs), and myeloid-derived suppressor cells (MDSCs). Extracellular matrix (ECM) and diverse secreted factors are also involved in shaping the CSC niche. The core of the tumor has a highly hypoxic environment. All these components coordinate to support CSC self-renewal and sustain tumor malignancy.

Collectively, the CSC niche represents a complex and adaptive ecosystem in which immune cells, stromal cells, and ECM components cooperatively regulate CSC function. These interactions not only maintain stemness and promote tumor progression but also establish a protective environment that limits therapeutic efficacy. A deeper understanding of these intricate relationships is essential for developing strategies aimed at disrupting the CSC niche and improving cancer treatment outcomes. A graphical depiction of the CSC niche within the TME is presented in Figure 1.

## 4. Emerging Immunotherapies Targeting CSCs in the TME

Immunotherapy is a therapeutic approach that harnesses the host’s immune system to generate an anti-tumor immune response, ultimately aiming to suppress or eliminate cancer [264]. The concept of immunotherapy emerged from studies showing that both innate and adaptive immune responses can recognize and eliminate malignant cells [265,266]. However, it is also understood that by the time of cancer diagnosis, malignant cells have often undergone immunoselection to evade immune-mediated tumor elimination. As a result, modern immunotherapy strategies aim not only to generate cytotoxic T cells but also to stimulate innate immunity [264].

**Figure 2:**
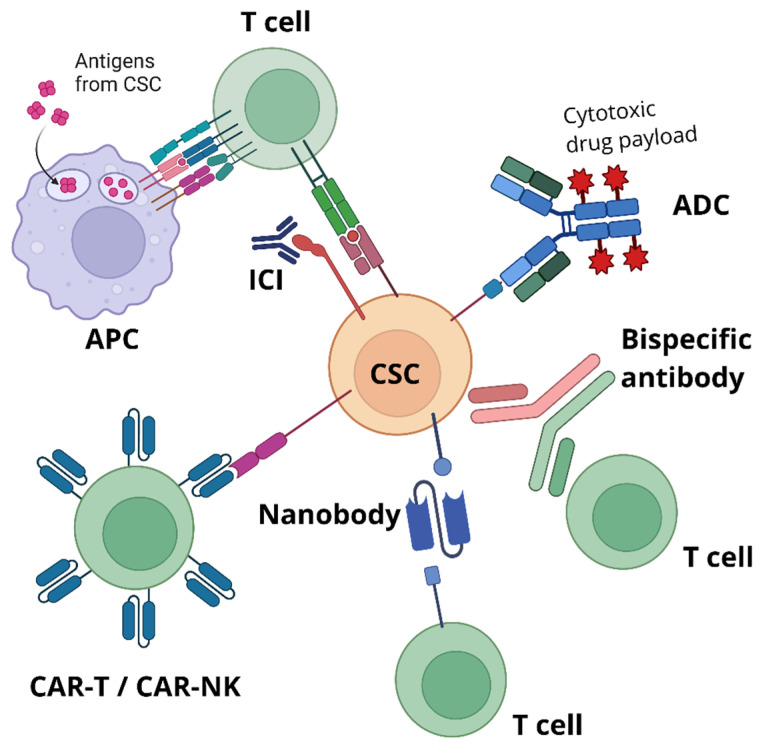
Immunotherapeutic strategies target cancer stem cells in the tumor microenvironment. Antigen-presenting cells (APCs) process and present CSC-derived antigens to T cells, initiating adaptive immune responses. Immune checkpoint inhibitors (ICIs) enhance T cell–mediated recognition and killing of CSCs by relieving inhibitory signaling pathways. Antibody-based strategies include antibody-drug conjugates (ADCs), which deliver cytotoxic payloads specifically to CSCs, and bispecific antibodies that simultaneously engage CSC-associated antigens and T cells to promote targeted cytotoxicity. Nanobodies provide smaller, high-affinity targeting agents with improved tumor penetration. Adoptive cell therapies such as chimeric antigen receptor T cells (CAR-T) and CAR-NK cells are engineered to recognize CSC-specific markers and directly eliminate CSC populations.

Current immunotherapies are categorized based on their mechanism of action into two main types: active immunotherapy and passive immunotherapy [267]. Active immunotherapy stimulates the patient’s own immune system to exert anti-tumor effects [267]. Examples of active immunotherapies include cancer vaccines [268] and immune checkpoint inhibitors (ICIs) [269], as well as Oncolytic virus (OVs) therapy. In contrast, passive immunotherapy involves the direct administration of immune-modulating agents to achieve anti-cancer effects. While this approach provides rapid and short-acting effects, it lacks sustained immunity [267]. Examples include antibody-drug conjugates (ADCs) and bispecific antibodies (BsAbs), both of which are monoclonal antibody (mAb)-based therapies [270], as well as cell-based therapies like chimeric antigen receptor T cell (CAR-T) and chimeric antigen receptor NK cell (CAR-NK) therapies [271]. We highlight the emerging therapeutic approach targeting CSCs in Figure 2.

In this section, we will explore the mechanisms of action, preclinical and/or clinical applications, and limitations of current immunotherapy targeting CSCs.

### 4.1. Immune Checkpoint Inhibitors

Immune checkpoints are cell-surface receptors expressed on various cell types, in particular immune cells and cancer cells. Examples of immune checkpoints include PD-1, CTLA-4, LAG3, TIM3, TIGIT, and BTLA. When these immune checkpoints bind to their respective ligands, they signal immune cells, particularly T cells, to stop their attack. Therefore, immune checkpoints are key regulators of immune response, helping to maintain self-tolerance and prevent autoimmune reactions [272]. However, in malignant cells and tumor-infiltrating myeloid cells, the ligands for these immune checkpoints are often expressed, which helps protect tumors from immune attacks. If these immunosuppressive signals persist over time, they can impair T cell functionality, reducing their ability to eliminate malignant cells. This phenomenon, known as “T cell exhaustion” [273], results in a weakened anti-tumor immune response. ICIs were developed to combat T cell exhaustion by reactivating T cell-mediated anti-tumor response. An FDA-approved ICIs, PD-1 inhibitors, have shown dramatic improvements in patient outcomes in clinical trials across various cancer types, particularly non-small cell lung cancer [274] and melanoma [275]. ICIs have now become a pillar of standard care for cancer patients and are often combined with various anti-cancer therapeutics to synergize efficacy.

Interestingly, in CSCs, PD-L1, the counterpart of PD-1, plays a role in maintaining stemness in breast cancer by regulating the expression of the transcription factors related to embryonic stem cells like OCT4A, NANOG, and BMI1. When PD-L1 is downregulated, the self-renewal capability of breast CSCs is significantly impaired [276]. Similarly, the interaction between PD-1 and PD-L1 has been shown to promote the survival of gastric CSCs [277]. A protective role for another immune checkpoint, CTLA-4, in supporting melanoma CSCs has also been observed [278]. These findings suggest that ICIs may serve as a promising therapeutic strategy for targeting CSCs in the TME. However, contradictory evidence indicates that immunotherapy might induce CSC phenotypes, therapy resistance, and metastasis in breast cancer cells. This occurs via T cell-mediated IFN-γ signaling and branched-chain amino acid aminotransaminase 1 (BCAT1) pathway. In this study, the authors further investigated the connection between anti-PD1 treatment, CSCs, and activation of the BCAT1 pathway in patient samples. Comparing patient data before and after anti-PD1 treatment revealed a significant increase in the percentage of CSCs, activation of the BCAT1 pathway, and expression of CSC-related genes [279]. These findings highlight the complexity in targeting CSCs using immunotherapy in the TME. Further investigations are needed to unravel the mechanisms of action of ICIs in CSCs and their broader effects within the TME.

### 4.2. Cancer Vaccines

Cancer vaccines are a strategy to enhance anti-cancer effects by leveraging tumor antigen presentation, which activates antigen-specific T cell-mediated killing of cancer cells [280]. Once the vaccine is injected into patients, APCs are recruited to the injection site, where they capture antigens and present them to naïve T cells via MHC class I, and to DCs via MHC class II in the lymph nodes. This process primes and expands the T cells, which are then released into the bloodstream and migrate to the TME to eliminate the tumor cells [281].

As a choice of antigen, traditional cancer vaccines typically use TAAs, which are expressed at higher levels in tumor cells relative to normal cells. However, the lack of specificity for cancer cells resulted in weak efficacy [282]. A more specific target for cancer vaccines is tumor-specific antigens (TSAs) or neoantigens, which are only found in cancer cells. TSAs are produced due to transcriptional dysregulation, such as alternative RNA splicing, genomic mutation, or altered post-translational modification [283]. Another emerging technology in cancer vaccine development is mRNA-loaded cell-based vaccines. Instead of directly injecting antigens like TAAs or TSAs, mRNA vaccines introduce mRNA that encodes the full-length target protein sequence into APCs. Inside the APCs, ribosomes translate the mRNA into the target protein, which is presented on MHC class I molecules [284]. When DCs function as APCs, the produced full-length proteins may be secreted or released after cell death into the TME, where nearby APCs can take up and present the antigen via MHC class II. Also, the immune response triggered by the vaccine depends on the type of antigen used. While both TAAs and TSAs activate CD4+ and CD8+ T cells, mRNA vaccines primarily induce a CD8+ T cell-mediated immune response [285].

In cancer vaccine targeting CSCs, DCs are typically the APCs used [286]. DC-based cancer vaccines targeting CSCs have shown promise in suppressing local recurrence, metastasis, and improving overall survival [287,288,289]. When two types of DC-based cancer vaccines were compared (one vaccine targeting glioma CSCs and the other one targeting glioma non-CSCs), the vaccine targeting CSCs resulted in higher tumor filtration of CD8+ and CD4+ T lymphocytes and provided better protection against glioma [287]. Another study highlighted the potential of DC-based cancer vaccine as a new adjuvant therapy combined with radiotherapy or surgical resection. In this study, when an ALDH^high^ D5 CSC-DC vaccine was administered after localized radiation, it significantly inhibited tumor growth, reduced lung metastasis, and prolonged survival in mice [288]. These promising preclinical results paved the way for clinical trials. In a phase I/II study, seven glioblastoma patients were treated with DCs pulsed with autologous glioma CSCs antigens. All seven patients demonstrated an immune response induced by the vaccine. Compared with matched controls, the immunized group showed 2.9-fold longer progression-free survival (median 694 days vs. 2236 days; *p*-value = 0.0018) (NCT00846456) [290]. While glioma CSC-DC vaccination appears promising, larger-scale trials are needed to confirm its efficacy. Additionally, several phase I/II studies targeting CSCs in other cancers, including lung CSCs (NCT02084823) [291], nasopharyngeal CSCs (NCT02115958) [292], and pancreatic CSCs (NCT02074046) have been conducted [293]. All three trials showed CSC-specific and CSC-nonspecific immune responses along with tolerable safety profiles. Moreover, a phase II trial of cancer vaccine (STEMVAC) targeting multiple antigens expressed in breast CSCs is currently active (NCT05455658).

DC-based cancer vaccines face several major challenges, ranging from effective processing and presentation of antigens in DCs to primed T cells to exert anti-tumor effects within the TME. The immunosuppressive nature of TME can impair antigen processing and presentation in cancer patients, affecting DC activation, migration to lymph nodes, and presentation of antigens to naïve T cells [294]. Additionally, even when naïve T cells are primed, their ability to egress from lymph nodes and traffic to the tumor site to kill malignant cells is often hindered by the immunosuppressive TME [295]. As a result, efforts have been made to combine DC-based cancer vaccines with ICIs, which can reverse the immunosuppressive TME into an immune-competent TME. These combinations have shown significant reductions in tumor relapses and prolonged survival [289]. Although not targeting CSCs, the phase IIb KEYNOTE-942 clinical trial, which tested mRNA-based cancer vaccines combined with ICIs, demonstrated improved recurrence-free survival compared to ICIs alone in high-risk melanoma (NCT03897881). Additionally, the mRNA-derived IDO/PD-L1-targeted vaccine (mRNA-4359), which elicits T cell activity against cancer and immune-suppressive cells expressing IDO1 and PD-L1, is currently undergoing a phase I/II trial (NCT05533697). Another challenge to effective T cell-mediated anti-tumor efficacy is stromal cells surrounding the tumor, which impede T cell infiltration into the TME and hinder tumor cell elimination [296]. To fully unlock the potential of cancer vaccines, these obstacles need to be overcome and addressing them will be crucial in future studies.

### 4.3. Monoclonal Antibodies, Nanobody and Bispecific Antibodies Strategy

mAbs are laboratory-engineered immunoglobulins designed to bind to specific antigens that are either unique to or overexpressed in tumors [297]. These antibodies exhibit a multifaceted anti-tumor mechanism of action, with the primary effect being the blockade of growth factor receptor signaling pathways, leading to the induction of tumor cell death [298]. Additionally, mAbs possess immunotherapeutic properties, similar to native antibodies like IgG, IgM, IgA, or IgE, by engaging immune cells within the surrounding TME. Their immunotherapeutic mechanisms include antibody-dependent cellular cytotoxicity (ADCC), complement-dependent cytotoxicity (CDC), and antibody-dependent cellular phagocytosis (ADCP). In ADCC, the Fc portion of mAbs binds to Fc receptors (FcRs) on NK cells, enabling these cells to target and destroy tumor cells. This mechanism is particularly effective against CSCs, which often evade immune surveillance by downregulating MHC class I molecules. NK cells are known to attack cells with reduced MHC class I expression, and mAbs can enhance this effect by tagging CSCs through CSC-specific surface antigens such as CD44, its splicing variant CD44v6 [267], EpCAM [299], and endoglin [300]. The expression of FcR-γ on immune effector cells is also crucial for ADCC efficacy; mutant FcR-γ in effector cells has been shown to result in a lack of therapeutic effect in vivo [301,302]. When the Fc region of mAbs binds to macrophages, ADCP occurs. While ADCP plays a vital role in targeting circulating tumor cells [303], its role in targeting CSCs within the TME remains largely unexplored. RG7356, a humanized anti-CD44 antibody, was evaluated in a phase I clinical trial for acute myeloid leukemia (NCT01641250) [304] and solid tumor (NCT01358903) [305], demonstrating good tolerability in both studies. Another phase II/III trial tested the trifunctional antibody catumaxomab (anti-EpCAM x anti-CD3) in malignant ascites patients (NCT00836654). An in vitro study showed that catumaxomab eliminated cancer cells by inducing the release of proinflammatory Th1 cytokines. Patient samples from pre-treatment and post-treatment demonstrated a greater than two-fold increase in CD4+ T cells and CD8+ T cell activation, alongside the elimination of CD133+/EpCAM+ CSCs [306].

Although mAbs hold promise as an immunotherapeutic strategy to target CSCs, antibody delivery remains one of the biggest challenges due to the complex structure of the TME. Traditional mAbs face several issues, including antibody instability, low bioavailability, and off-target effects [307]. To overcome these challenges while preserving the immunological effects of the antibody, nanobodies have emerged as a promising solution. Nanobodies are single-domain antibodies composed solely of recombinant variable domains of the heavy chain. Due to their small size, high solubility, high stability, deep tissue penetration and rapid clearance from the bloodstream, nanobodies are expected to provide improved diagnostic and therapeutic tools [308]. For example, a nanobody targeting mitochondrial translation elongation factor (TUFM) has been applied to target glioblastoma stem cells. The anti-TUFM nanobody (Nb206) demonstrated specificity and potent inhibitory effect on the growth of glioblastoma stem cells in vitro [309]. The effect of Nb206 in a clinical setting has not yet been examined. Similarly, the anti-TRIM28 nanobody (NB237) showed significant inhibition of glioma CSCs’ invasiveness and growth in an in vivo model [310]. Another example is an anti-HER2 nanobody conjugated to polyamidoamine (PAMAM) dendrimers, designed to target breast CSCs. The nanobody first binds to HER2 receptors, which are overexpressed on breast CSCs, facilitating the cellular uptake of the PAMAM dendrimers. Once inside the cell, PAMAM dendrimer induces transcription of apoptosis-inducing tBid expression. In vitro testing of this nanobody demonstrated efficient induction of apoptosis in breast CSCs [311].

BsAbs are another emerging antibody-based therapeutic approach. BsAbs are engineered immunotherapeutics designed to simultaneously bind two distinct antigens, typically one expressed on CSCs and another on immune effector cells such as T cells [312]. Most commonly, these antibodies engage a CSC-associated surface marker such as CD133 or EpCAM and CD3 on T cells, thereby physically bridging immune cells to CSCs [313,314]. This proximity promotes T cell activation, immune synapse formation, and targeted cytotoxicity against CSCs independent of peptide-MHC recognition [315]. By directly redirecting immune effector function toward CSC populations, BsAbs aim to eliminate the subpopulation of tumor cells responsible for tumor initiation, metastasis, and therapeutic resistance. Preclinical studies have demonstrated promising results for CSC-targeting BsAbs in both in vitro and in vivo settings. For example, EpCAM/CD3 BsAbs (MT110) have been shown to effectively redirect T cells to eliminate EpCAM^+^ CSCs in pancreatic cancer [316]. Similarly, CD133/CD3 BsAbs have demonstrated potent cytotoxic activity against CSC-enriched populations in colorectal cancer and glioblastoma. In vivo, these approaches have led to reduced tumor growth and delayed recurrence in xenograft mouse models, supporting the idea that targeting CSCs can improve long-term tumor control compared to therapies that primarily eliminate bulk tumor cells [317,318]. Clinically, several bispecific antibody platforms have entered early-phase trials, although relatively few are exclusively designed for CSC-specific targets. Some CD3-engaging BsAbs targeting antigens enriched in stem-like tumor populations, such as EpCAM (MT701), have been evaluated in patients with solid tumors (NCT06266091), demonstrating manageable safety profiles and preliminary signs of efficacy [319]. A phase III trial of anti-EpCAM x anti-CD3 bispecific antibody MT701 is ongoing (NCT06432296). Additionally, ongoing clinical development of bispecific antibodies in oncology more broadly, particularly those targeting TAAs alongside immune effector molecules, provides a strong foundation for extending this strategy to CSC-specific markers. As our understanding of CSC biology and surface marker specificity improves, BsAbs are expected to become an increasingly precise and effective tool for eradicating tumor-initiating cells and preventing relapse.

### 4.4. Antibody-Drug Conjugates

ADCs represent another class of mAb-based therapies. As the name suggests, ADCs consist of mAbs conjugated with chemotherapeutic agents or radioactive isotopes. ADCs comprise three main components: an IgG antibody, a payload (a drug of choice), and a linker that connects the antibody to the drug. In addition to the mechanism of action inherent from mAbs, ADCs exert anti-tumor effects through the activity of their conjugated payloads. When the antibody portion of an ADC binds to its target antigen, the complex is internalized via endocytosis. Inside the cell, the ADC is transported to the lysosome, where the acidic environment cleaves the linker, releasing the payload and inducing cell death [270]. This mechanism allows ADCs to deliver drugs selectively to tumors, potentially reducing systemic side effects. However, non-specific release of the payload, or “off-target effects,” has been observed in clinical trials. These effects, caused by the instability of cleavable linkers in circulation, were first identified through retrospective reviews of second-generation ADC trials [320,321]. As research into the mechanism of action for ADCs has advanced, an intriguing phenomenon known as the “bystander effect” has been identified [322]. The effect occurs when the payload of ADC diffuses out of the targeted cell and into neighboring cells. Tumor within the TME is often heterogeneous, with uneven antigen expression, which can limit ADCs’ efficacy. However, the bystander effect helps overcome this challenge [323]. One potential real-life example of this bystander effect is trastuzumab-deruxtecan (T-Dxd), a HER2-targeting ADC. T-Dxd has demonstrated significant survival benefits in patients with HER2-low and HER2-ultra-low metastatic breast cancer [324].

Targeting CSCs with ADCs involves designing the antibody portion to recognize antigens that are uniquely or highly expressed in CSCs, similar to the approach used for mAbs. One such ADC, bivatuzumab mertansine, consists of an anti-CD44v6 antibody covalently linked to the cytotoxic agent mertansine. Unfortunately, its clinical development was halted during a phase I trial due to a severe skin toxicity profile before reaching the maximum tolerated dose [325].

### 4.5. Cell-Based Immunotherapy: CAR-T and CAR-NK Cell Therapies

CAR-T cell therapy is a form of personalized cellular immunotherapy widely used to treat hematological cancers [326]. CARs are synthetically engineered receptors designed to enable T cells to recognize and eliminate cells expressing specific antigens. To create CAR-T cells, a patient’s T cells are collected and genetically modified in the laboratory to express CARs on their surface. The CAR-T cells are then infused back into the patient. CARs are specifically designed to recognize and bind to antigens expressed on malignant cells. The internal domain of a CAR consists of signaling components that activate the T cell upon binding. When the external portion of the CAR binds to a target antigen on cancer cells, the internal domain transmits signals that trigger the T cell to attack and destroy the cancer cell [327].

Unlike normal T cell receptors, which require antigens to be presented by MHC molecules, CARs can bind the target antigen independently of MHC molecules [328]. This feature makes CAR-T cells particularly advantageous for targeting CSCs, as CSCs often exhibit reduced expression of MHC molecules [329]. As a result, efforts have been made to develop CAR-T cells capable of recognizing and binding to surface markers highly expressed in CSCs. Examples of these markers include CD22, CD124, ALDH, CD133, CD57, epidermal growth factor receptor variant III (EGFRvIII) [267]. For instance, anti-CD133 CAR-T cell therapy has shown promise in targeting glioblastoma. In both in vitro and in vivo, anti-CD133 CAR-T cells effectively killed CD133^+^ glioma stem cells. However, they failed to eradicate glioblastoma entirely due to the tumor’s induction of terminal differentiation [330]. Similarly, anti-EGFRvIII CAR-T cells demonstrated effector cytokine production, including IFN-γ, and successful lysis of target cells in vitro [331]. This anti-EGFRvIII CAR-T cell therapy was further evaluated in a phase I clinical trial involving 10 recurrent glioblastoma patients (NCT02209376). One patient showed residual stable disease for over 18 months of follow-up. Among 10 patients infused with CAR-T cells, 7 underwent post-CAR-T surgical interventions, allowing for in situ analysis of tumor site specimens. The analysis revealed that anti-EGFRvIII CAR-T cells successfully trafficked to the tumor site. However, tumor antigen expression decreased, immune inhibitory molecules increased, and regulatory T cells infiltrated into the active tumor site, potentially limiting therapeutic efficacy [332].

As this study suggests, CAR-T therapy has not demonstrated robust efficacy in solid tumors as it has in hematological malignancies. Despite more than 700 clinical trials testing CAR-T cell therapy in solid tumors, none have received FDA approval [326]. One key reason for this limited efficacy is immunosuppressive TME. In solid tumors, cells within the TME, such as TAMs, Tregs, MDSCs, and CAFs, can directly impair CAR-T cell function and foster a pro-tumor environment [333]. Additionally, cytokines, chemokines, and growth factors secreted within the TME contribute to CAR-T cell dysfunction and the recruitment of suppressive immune cells [334]. T cell exhaustion caused by co-inhibitory pathways further reduces CAR-T cell efficacy. A combination of ICIs with CAR-T cell therapy in glioma models, including murine and canine, has been shown to successfully reverse T cell anergy [335]. Also, a phase I clinical trial (NCT02414269) testing CAR-T cell therapy combined with ICIs in 27 patients with malignant pleural mesothelioma also showed encouraging results. Among the 27 patients who received CAR-T cell therapy, 18 patients received ICIs followed by CAR-T cell therapy. Of these, eight patients experienced stable disease sustained for more than 6 months, and two patients achieved complete metabolic response as shown by positron emission tomography (PET) scan [336]. While these findings suggest that CAR-T cell therapy may have potential in solid tumors, the study was limited to phase I, highlighting the need for further research to validate the effectiveness of combining CAR-T cells with ICIs. Beyond this approach, additional strategies are required to enhance CAR-T cell function in the complex TME. Although CAR-T cell therapy has shown remarkable efficacy in hematological cancers, overcoming the unique challenges of the TME in solid tumors will require innovative combination therapies and novel immunotherapeutic approaches [327]. One such example of combination therapy will be discussed in Section 5.1 Targeted Protein Degradation.

CAR-NK cell therapy is an emerging form of cell-mediated immunotherapy. Similar to CAR-T cells, CAR-NK cells are NK cells that are genetically modified in the laboratory to express CARs. These receptors enable CAR-NK cells to recognize specific tumor surface proteins and activate signaling pathways that enhance their ability to kill tumor cells [337]. While CAR-NK cell therapy is a relatively new concept, most studies to date have focused on hematological cancers, and its efficacy in targeting CSCs requires further investigation [337]. One promising approach is the use of anti-EpCAM CAR-NK cell therapy. EpCAM, a marker associated with CSCs and known for promoting cancer cell stemness [338,339], has been targeted in preclinical studies. In colon cancer models, anti-EpCAM CAR-NK cells demonstrated the ability to specifically recognize EpCAM+ colon cancer cells, release effector cytokines such as perforin and granzyme B, and exert significant anti-tumor effects in vitro [340].

Despite these encouraging results, CAR-NK cell therapy faces challenges similar to those of CAR-T cells, particularly in the immunosuppressive TME, and the mechanisms of which are largely unknown [341]. Factors such as hypoxia and altered tumor metabolism can impair CAR-NK cell function [337]. For instance, under hypoxic conditions, reduced phosphorylation of ERK and STAT3 inhibits the activation of CAR-NK cell receptors, leading to diminished cytotoxicity [342]. Hypoxia also upregulates the expression of stemness markers like NANOG, SOX2, and OCT4 in CSCs, potentially exacerbating the challenge [343]. Additionally, the glycolytic metabolism of tumors creates a low-pH environment due to lactic acid production from glucose. This acidic TME inhibits NFAT expression, reducing CAR-NK cells’ production of IFN-γ and further compromising their efficacy [344]. Future research should aim to address these limitations by optimizing CAR-NK cell designs and developing strategies to modulate the TME. Advancements in these areas could significantly enhance the therapeutic potential of CAR-NK cells for targeting CSCs and solid tumors.

### 4.6. Oncolytic Viruses and Immune Modulators

OVs are engineered viruses that have tropism to cancer cells and the ability to lyse them. OV-mediated therapies have been approved as one of the cancer immunotherapies, with examples including Rigvir (ECHO-7) and T-VEC (Imlygic) for melanoma [345,346], Oncorine (H101) for head and neck cancer [347], and DELYTACT (teserpaturev/G47delta) for glioblastoma [348]. OVs exert anti-tumor effects through mechanisms like natural viral infections, involving selective infection of tumor cells, replication within the tumor, lysis of tumor cells, and activation of anti-tumor immunity [349]. To enhance their cytotoxic effect, OVs are often armed with immune-stimulating eukaryotic transgene payloads, foreign genes inserted into the OV DNA sequence [350]. For instance, T-VEC encodes granulocyte-macrophage colony-stimulating factor (GM-CSF) to enhance the immune responses [346]. Achieving cancer cell specificity, or oncotropism, requires consideration of factors such as cell surface receptors that facilitate viral entry, cancer cell metabolism, and the ability of OVs to evade innate antiviral responses within cancer cells [351]. Interestingly, the TME often provides favorable conditions for OV replication due to compromised antiviral immune responses [352]. This is particularly true in tumors with activated stemness programs, where pathways like type I IFN signaling are repressed. Such environments, enriched with CSCs, may further promote OV replication, though this requires more investigation [353]. Despite their ability to evade innate antiviral responses, OVs face immunological barriers, including preexisting neutralizing antibodies and adaptive immune responses, which can indirectly inhibit their function [354]. Additionally, some OVs, such as the Maraba virus [355] and oncolytic Newcastle disease virus [356], may induce upregulation of the PD-1/PD-L1 axis on tumor and immune cells in the TME, potentially limiting their therapeutic efficacy.

Application of OVs in eradicating CSCs is a suitable therapeutic approach for several reasons. CSCs are highly resistant to chemotherapy and radiation therapy, often leading to recurrence or metastasis at a later stage of the disease progression, but OVs provide a novel approach to effectively target these resistant cells [357,358]. For example, oncolytic vaccinia virus (VACV) GLV-1h68 selectively proliferated in and eradicated cancer stem-like cells resistant to chemotherapy and/or radiation therapy in breast cancer xenograft models [359]. Similarly, cancer-favoring oncolytic vaccinia virus (CVV) demonstrated successful CSC killing and overcame chemotherapy resistance in human colon cancer cell lines. CVV has characteristics of favoring cancer and a different cytotoxic pathway for killing tumors. CVV’s unique cancer-targeting properties and distinct cytotoxic pathways enabled it to eliminate colon cancer cells regardless of stem cell-like colon cancer cells presence [360].

Secondly, OVs can be engineered to produce cytotoxic proteins through transgenes that specifically target CSC-related pathways, such as those involved in self-renewal and differentiation [349]. Many OVs in clinical and preclinical trials are designed to carry immunomodulatory transgenes like GM-CSF and intercellular adhesion molecules (ICAM-1) to enhance immune responses [361,362]. OVs not only directly kill malignant cells through cell lysis but also via activating the host’s immune response, potentially transforming the TME from “immune-cold” to “immune-hot” [349]. Another example of a non-immunomodulatory transgene is the herpes simplex virus (HSV) engineered with the TK suicide gene to target CD133, a CSC marker. This approach significantly reduced proliferation and induced apoptosis in liver CSCs in vitro [363]. Clinical trials have also demonstrated the potential of OVs to induce immunogenic cell death. For instance, in the phase I clinical trial (NCT00805376), 37 patients with recurrent glioma received DNX-2401 (Delta-24-RGD; tasadenoturev) intratumorally, leading to immune activation. Post-treatment species revealed the induction of immunogenic cell death, but PD-1 expression remained the same [364]. Thus, the combination of DNX-2401 and anti-PD-1 antibodies was tested in 49 recurrent glioblastoma patients in a phase I/II trial, but only selective patients benefited from this treatment (NCT02798406) [365].

Another key advantage of targeting CSCs with OV-mediated vaccine is the potential of preventing disease recurrence. A study explored this approach using a bovine herpesvirus 4 (BoHV-4) vector to generate immune memory against the cystine-glutamate antiporter xCT (SLC7A11), a protein overexpressed in mammary CSCs. SLC7A11 plays a crucial role in maintaining CSCs’ redox balance, self-renewal, and resistance to chemotherapy. To develop an anti-xCT viral vaccine, xCT was expressed in the BoHV-4 vector (BoHV-4-mxCT), which was then used to infect mice inoculated with breast cancer cell lines prone to metastasis. Mouse infected with BoHV-4-mxCT revealed less lung metastasis, along with the evidence of T cell activation, anti-xCT antibody formation, and ADCC [366]. Although not targeting CSCs, several viral vector-based personalized vaccines are also being tested in clinical trials targeting metastatic melanoma (NOUS-PEV; NCT04990479/EVX-01; NCT03715985) [367,368], and solid tumor (NCT03568058). For instance, the NOUS-PEV study demonstrated the induction of targeted neoantigen-specific immune response and the expansion of diverse T cell receptor (TCR) clonotypes in the post-treatment biopsied samples [367]. These preclinical and clinical findings highlight the potential of OVs as a therapeutic tool for curing metastatic disease. However, further research is needed to develop vaccines targeting CSC-specific antigens [366].

The recent clinical trials of immunotherapies targeting CSCs are shown in Table 2.

## 5. Innovative Strategies and Future Directions

### 5.1. Targeted Protein Degradation

Targeted protein degradation (TPD) is an emerging immunotherapeutic modality that selectively degrades specific proteins of interest. One of the primary tools enabling this approach is proteolysis-targeting chimeras (PROTACs). PROTACs are bifunctional small molecules composed of two ligands connected by a linker; one ligand binds to the protein of interest (POI), targeting POI for degradation, while the other ligand recruits an E3 ubiquitin ligase, promoting POI ubiquitination and subsequent proteasomal degradation [369].

PROTACs offer significant potential because they can target previously “undruggable” proteins, including certain transcription factors [370]. However, studies exploring PROTACs in targeting CSCs via immune responses remain limited. Recently, PROTACs targeting immune-related molecules in cancer therapy have started to gain attention. Multiple PROTACs are designed to target PD-1/PD-L1 proteins, including compound 22, AC-1, AbTACs, CDTAC, compound 21a, peptide-PROTACs, R2PD1, SP-PROTAC, and linear peptide PROTAC. These compounds inhibit immune checkpoint signaling through post-translational modifications such as phosphorylation, acetylation, palmitoylation, glycosylation, and UFMylation [371]. An in vivo study showed that compound 21a increased CD8+ T cells infiltration and decreased murine colon cancer growth by lowering PD-L1 protein expression [372]. Thus, by targeting immunomodulatory proteins, PROTACs may help reverse the immunosuppressive TME.

Beyond immunotherapy, PROTACs may also target CSCs indirectly by modulating epigenetic regulators. Epigenetic dysregulation contributes to stemness and self-renewal in CSCs [373]. PROTACs targeting epigenetic modifications, such as histone acetylation, histone methylation, and chromatin remodeling, hold potential for CSC therapy [374]. Further studies are expected to evaluate PROTAC-mediated epigenetic therapy targeting CSCs. Additionally, miRNA-based PROTACs have emerged as a novel approach to target CSCs. For example, Lin28A-miRNA-PROTAC was designed to degrade the protein Lin28A, increasing tumor-suppressive let-7 miRNA levels (target of Lin28A). Lin28 is an RNA-binding protein and a carcinogenic protein that results in the generation of CSCs. In breast cancer models, Lin28A-miRNA-PROTAC induced apoptosis and increased chemotherapy sensitivity. Also, when combined with tamoxifen, it significantly regressed the tumor in in vivo models [375].

In addition, combination strategies incorporating PROTAC-based approaches with other therapeutic modalities are likely to provide synergistic benefits and should be further explored. PROTAC technologies offer a promising means to modulate these epigenetic constraints on T cell function, where epigenetic regulation plays a central role in T cell exhaustion and limits the efficacy of T cell-based immunotherapies [376]. For example, targeting epigenetic regulators such as EZH2, using inhibitors like tazemetostat, is being investigated to enhance antitumor immunity, in part by reducing Treg-mediated immunosuppression [377]. CAR-T cells are particularly susceptible to exhaustion, partly due to tonic signaling from the synthetic receptor even in the absence of antigen. In this context, transient attenuation of such signaling has been shown to restore T cell functionality, with evidence suggesting that this re-gain of function is mediated through EZH2-dependent epigenetic remodeling [378]. Another important limitation of T cell–mediated therapies is the inefficient infiltration of T cells into tumor sites, which is often hindered by the presence of a dense ECM [379]. This physical barrier restricts immune cell access and contributes to reduced therapeutic efficacy. Recent studies suggest that targeted degradation of discoidin domain receptor 1 (DDR1), a collagen-binding receptor involved in the ECM organization, using PROTAC technology may help overcome this barrier. By disrupting DDR1-mediated matrix alignment and stiffness, PROTAC-driven DDR1 degradation can enhance T cell penetration into tumors. Therefore, combining DDR1-targeting PROTACs with T cell-based therapies may represent another promising strategy to improve immune infiltration and overall antitumor efficacy [380]. These advancements could unlock new therapeutic possibilities in cancer treatment.

### 5.2. Future Directions

CSCs play a pivotal role in cancer recurrence, metastasis, resistance to conventional treatment, and tumor heterogeneity, making them challenging targets for therapy [268]. Fortunately, recent advances in cancer immunotherapy have opened new avenues for targeting CSCs, with several studies demonstrating promising results in eliminating these resilient cells [336].

Despite these advancements, significant challenges remain. One major obstacle is the immunosuppressive TME. Since most immunotherapies rely on the host’s immune system activation, the suppressive nature of the TME often hampers their efficacy. To address this issue, researchers are exploring combination strategies that integrate multiple therapeutic approaches to leverage their synergistic effect. For example, ICIs are frequently combined not only with other immunotherapies but also with radiation therapy, chemotherapy, and small-molecule inhibitors. These combinations aim to modulate the TME and enhance overall therapeutic efficacy, with several studies reporting encouraging outcomes. Further investigation of combination strategies is needed to overcome the limitations of individual therapies.

Another significant challenge is identifying CSC-specific antigens. While antibody-mediated immunotherapies have demonstrated robust efficacy in targeting specific cancer types and have become a standard care in some cases, the development of CSC-specific therapies remains an area of active investigation. Additionally, clinical trials evaluating the long-term survival benefits and overall efficacy of CSC-targeted immunotherapies are still needed.

**Figure 3:**
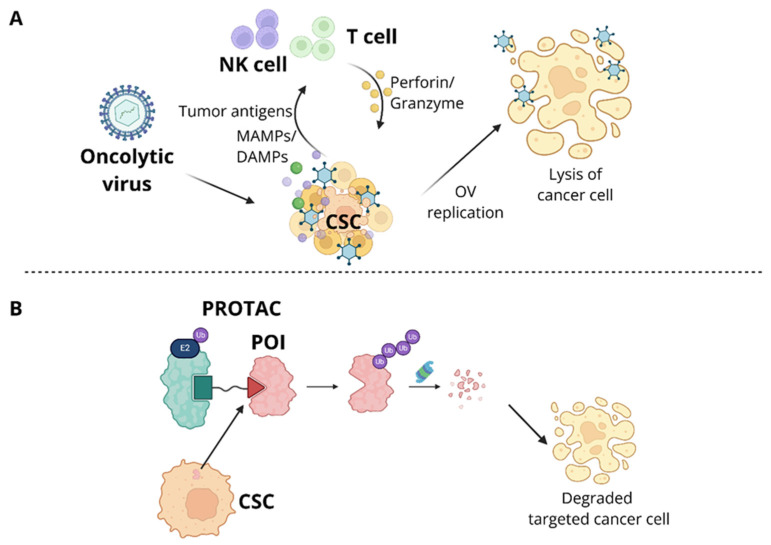
Innovative therapeutic strategies targeting cancer stem cells (CSCs): oncolytic virotherapy and targeted protein degradation. (**A**) Oncolytic virus (OV)-mediated targeting of CSCs. Oncolytic viruses selectively infect and replicate within CSCs, leading to direct lysis of cancer cells. OV infection triggers the release of tumor-associated antigens, microbe-associated molecular patterns (MAMPs) and damage-associated molecular patterns (DAMPs), which enhance immune activation. These signals recruit and activate both innate and adaptive immune cells, which further eliminate CSCs through cytotoxic mechanisms such as perforin and granzyme release. This dual mechanism of OV therapy direct tumor killing with a broader immune response against CSC. (**B**) PROTAC-mediated degradation of CSC-associated targets. Proteolysis-targeting chimeras (PROTACs) induce selective degradation of proteins of interest (POIs) critical for CSC maintenance. PROTAC molecules simultaneously bind the target protein and an E3 ubiquitin ligase, facilitating ubiquitination of the POI and its subsequent degradation via the proteasome. This targeted protein degradation disrupts key oncogenic signaling pathways in CSCs, ultimately leading to loss of CSC viability and tumor cell elimination.

Future research should focus on overcoming these challenges by advancing combination strategies and identifying novel CSC-specific antigens. Such efforts could pave the way for more effective and durable cancer therapies. We highlight innovative elimination-based therapies in Figure 3.

## 6. Conclusions

CSCs represent a critical therapeutic target due to their central roles in tumor initiation, progression, metastasis, and relapse. However, their effective elimination remains a major challenge, largely because of their intrinsic plasticity and the profound influence on the TME. The TME not only sustains CSC phenotypes through diverse biochemical and physical cues but also establishes a deeply immunosuppressive milieu that limits immune recognition and effector function. As a result, CSCs are able to evade immune surveillance and persist despite conventional therapies and, in many cases, immunotherapeutic interventions.

Recent advances in immunotherapy have provided promising strategies to target CSCs, including immune checkpoint blockade, CAR-T cell therapy, cancer vaccines, and antibody-based approaches. Several preclinical studies and early-phase clinical trials have demonstrated that CSC-associated antigens can be recognized by the immune system and exploited therapeutically. However, clinical outcomes have been variable, highlighting the challenges posed by CSC heterogeneity, antigen variability, and TME-mediated immune suppression. In particular, the presence of immunosuppressive cell populations, metabolic constraints, and physical barriers within the TME significantly limits immune cell infiltration and function, thereby reducing the efficiency of immunotherapies.

Importantly, emerging evidence suggests that successful targeting of CSCs will require combinatorial strategies that address both tumor-intrinsic properties and the surrounding microenvironment. Approaches aimed at reprogramming the TME, such as depletion of regulatory immune cells, inhibition of immunosuppressive signaling pathways, normalization of tumor vasculature, and modulation of metabolic conditions, may enhance immune-mediated CSC eradication. At the same time, the identification of more specific and stable CSC markers, along with advances in single-cell and spatial profiling technologies, will improve the precision of targeted therapies and patient stratification. These integrative strategies are likely to be essential for overcoming resistance and achieving durable therapeutic responses.

Ultimately, bridging the gap between mechanistic insights and clinical application will be key for advancing the field. A deeper understanding of the dynamic interplay between CSCs and the TME, combined with rationally designed clinical trials, holds significant promise for the development of more effective and lasting cancer treatments. By simultaneously targeting CSCs and the immunosuppressive networks that protect them, future immunotherapeutic approaches may overcome current limitations and lead to significant improvements in patient outcomes.

## Figures and Tables

**Table 1:** Table of each cancer type’s CSC markers.

Cancer Subtype	Common CSC Markers	Reference
Breast cancer	CD44^+^/CD24^−/low^ ALDH1^+^ EpCAM^+^/CD49f^−^ EpCAM^−^/CD49f^+^ CD133^+^	[58] [59] [60]
Colorectal cancer	CD133^+^ CD44v8-10^+^ ALDH1^+^ LGR5^+^ EpCAM^high^/CD44^+^	[61] [62] [63] [64] [65]
Lung cancer	CD133^+^ ALDH1^+^ CD44^+^ CD90^+^ SOX2^+^	[66] [67] [68] [69] [70]
Pancreatic cancer	CD44^+^ CD24^+^ ESA^+^ CD133^+^ ALDH1^+^	[71] [72] [10] [46] [73]
Glioblastoma	CD133^+^ SOX2^+^ Nestin^+^ CD15^+^ (SSEA-1) ALDH1^+^ A2B5 GD3	[74] [75] [76] [77] [78] [49] [48]
Ovarian cancer	CD133^+^ ALDH1^+^ CD44^+^ CD117^+^ EpCAM^+^	[79] [80] [81] [82]
Prostate cancer	CD44v8-10^+^ α2β1^high^ CD133^+^ ALDH1^+^ PSCA	[83] [84] [85] [86]
Head and neck cancer	CD44^+^ ALDH1^+^ CD133^+^ SOX2^+^	[87] [88] [89] [90]
Melanoma	CD271^+^ ALDH^+^ ABCB5^+^ CD133^+^	[91] [92] [93] [94]
Leukemia (AML)	CD34^+^CD38^−^ CD123^+^ CD96^+^ C-KIT^+^	[95] [96] [97] [98]
Hepatocellular carcinoma	CD133^+^ EpCAM^+^ CD90^+^ CD44^+^ ALDH1^+^	[99] [100] [101] [102] [103]
Gastric cancer	CD44^+^ CD133^+^ ALDH1^+^ LGR5^+^	[104] [105] [106] [107]
Esophageal cancer	CD44^+^ CD133^+^ ALDH1^+^	[108] [109]
Bladder cancer	CD44^+^ ALDH1^+^ CD133^+^	[110] [111] [112]
Cervical cancer	ALDH1^+^ CD44^+^ SOX2^+^	[113] [114]
Renal cell cancer	CD105^−^/CD44^−^ CXCR4^+^	[115] [116]
Thyroid cancer	CD44^+^ CD133^+^ SSEA-1^+^	[117] [118] [119]
Testicular germ cell tumors	OCT4^+^ SSEA-4^+^	[120] [121]
Multiple myeloma	CD138^−^/CD19^+^fraction CD27^+^	[122] [123]
Osteosarcoma	CD133^+^ ALDH1^+^ CD117^+^ STRO-1^+^	[124] [125] [126]
Mesothelioma	CD44^+^ ALDH1^+^ CD133^+^	[127] [128] [129]

**Table 2:** Ongoing and completed clinical trials targeting CSCs with immunotherapy.

Approach	Cancer Type	Phase	Registration Number
Combination with an immune checkpoint inhibitor	Melanoma	Phase I (active)	NCT03161431
	Wnt (+) tumors	Phase I (completed)	NCT01351103
	Advanced cancer	Phase I (completed)	NCT03568058
Cancer vaccine	Glioma	Phase I/II (completed)	NCT00846456
	Glioma	Phase II/III (recruiting)	NCT03548571
	Nasopharyngeal cancer	Phase I/II (completed)	NCT02115958
	Ovarian cancer	Phase I/II (completed)	NCT02178670
	Lung cancer	Phase I/II (completed)	NCT02084823
	Hepatocellular carcinoma	Phase I/II (completed)	NCT02089919
	Colorectal cancer	Phase I/II (completed)	NCT02176746
	Pancreatic cancer	Phase I/II (completed)	NCT02074046
Monoclonal antibody	CD44 (+) solid tumors	Phase I (completed)	NCT01358903
	AML	Phase I (completed)	NCT01641250
	Solid tumors	Phase I (completed)	NCT01345201
	Solid tumors	Phase I (completed)	NCT01778439
	Lymphoid malignancies	Phase I (completed)	NCT01703572
	Solid tumors	Phase I (completed)	NCT00871559
	NSCLC	Phase I (completed)	NCT01189968
	Pancreatic cancer	Phase I (completed)	NCT01189929
	Colorectal cancer	Phase I (completed)	NCT01189942
Antibody-drug conjugate	SCLC	Phase III (completed)	NCT03061812
	Triple-negative breast cancer	Phase I (completed)	NCT03243331
	Solid tumor	Phase I (completed)	NCT02222922
	Solid tumor	Phase I (terminated)	NCT02078752
	AML	Phase I (terminated)	NCT02848248
	Solid tumor	Phase I (terminated)	NCT01891669
	CD56 (+) solid tumor	Phase I (completed)	NCT00346385
CAR-T	Malignant glioma	Phase I (unknown)	NCT03423992
	Advanced malignancies	Phase I/II (completed)	NCT02541370
	CD44v6 (+) tumors	Phase I/II (unknown)	NCT04427449
	AML, MM	Phase I/II (terminated)	NCT04097301
	MM	Phase I (unknown)	NCT04727008
	ROR1 (+) tumors	Phase I (terminated)	NCT02706392
	Sarcoma	Phase I/II (unknown)	NCT03356782
	EpCAM (+) solid tumor	Phase I (unknown)	NCT02915445
	Hepatocellular carcinoma	Phase II (unknown)	NCT02729493
Oncolytic virus	Malignant gliomas	Phase I (completed)	NCT00805376
	Glioblastoma, gliosarcoma	Phase II (completed)	NCT02798406
	Solid tumors	Phase I/II (unknown)	NCT03715985
Immune modulator	Triple-negative breast cancer	Phase II (completed)	NCT02370238
	HER2 (−) breast cancer	Phase I (completed)	NCT02001974
	Triple-negative breast cancer	Phase II (active)	NCT05455658
Bispecific antibody	Solid tumors	Phase I (completed)	NCT00635596

## Data Availability

No new data were created or analyzed in this study.
